# Effect of CO_2_ Concentration on the Performance of Polymer-Enhanced Foam at the Steam Front

**DOI:** 10.3390/polym16192726

**Published:** 2024-09-26

**Authors:** Mingxuan Wu, Binfei Li, Liwei Ruan, Chao Zhang, Yongqiang Tang, Zhaomin Li

**Affiliations:** 1 Key Laboratory of Unconventional Oil & Gas Development, China University of Petroleum (East China), Ministry of Education, Qingdao 266580, China; 2 School of Petroleum Engineering, China University of Petroleum (East China), Qingdao 266580, China; 3 Sinopec Petroleum Exploration & Production Research Institute, Beijing 100083, China

**Keywords:** steam front, polymer-enhanced foam, CO_2_, foam stability

## Abstract

This study examines the impact of CO_2_ concentration on the stability and plugging performance of polymer-enhanced foam (PEF) under high-temperature and high-pressure conditions representative of the steam front in heavy oil reservoirs. Bulk foam experiments were conducted to analyze the foam performance, interfacial properties, and rheological behavior of CHSB surfactant and Z364 polymer in different CO_2_ and N_2_ gas environments. Additionally, core flooding experiments were performed to investigate the plugging performance of PEF in porous media and the factors influencing it. The results indicate that a reduction in CO_2_ concentration in the foam, due to the lower solubility of N_2_ in water and the reduced permeability of the liquid film, enhances foam stability and flow resistance in porous media. The addition of polymers was found to significantly improve the stability of the liquid film and the flow viscosity of the foam, particularly under high-temperature conditions, effectively mitigating the foam strength degradation caused by CO_2_ dissolution. However, at 200 °C, a notable decrease in foam stability and a sharp reduction in the resistance factor were observed. Overall, the study elucidates the effects of gas type, temperature, and polymer concentration on the flow and plugging performance of PEF in porous media, providing reference for fluid mobility control at the steam front in heavy oil recovery.

## 1. Introduction

The primary method for heavy oil reservoir exploitation is thermal recovery, such as steam injection, which reduces the viscosity of crude oil. However, the generation of steam requires substantial energy consumption [1,2]. To improve the effectiveness of steam-based recovery in heavy oil reservoirs, a series of new technologies have been developed, with multiphase thermal fluid extraction gaining significant attention. Due to the heterogeneous nature of reservoirs and the high mobility ratio between the displacing fluids (e.g., gas or water) and crude oil, as well as gravity segregation [3,4], injected steam and other high-temperature fluids often rapidly channel through high-permeability pathways with low oil saturation, limiting the enhancement in oil recovery [5,6].

Since the initial report on using foam to control gas flow in 1958 [7], injecting foam into reservoirs to manage fluid mobility has gained widespread interest [8,9,10]. Under reservoir conditions, the capillary forces in porous media significantly increase the foam flow resistance, causing the gas-laden foam to block pore spaces and substantially reduce the gas relative permeability [11]. This method of enhancing oil recovery shows considerable potential for widespread application [12]. Foam, a gas-liquid two-phase system, disperses gas within a liquid phase, separated by thin liquid films [13]. However, foam is thermodynamically unstable and undergoes irreversible decay over time [14]. Viscosity has a significant impact on bulk foam stability; generally, more viscous foams are more stable [15,16,17,18]. In most cases, it is sufficient for the gas–liquid interface to have a high viscosity, without requiring the bulk solution to be highly viscous [19]. Adding polymers to water-based foam systems to form polymer-enhanced foam is a common approach. The polymers in the foam can include any water-soluble thickening agents, such as xanthan gum, modified starch, polyvinyl alcohol, and polyacrylamide [19,20]. For foam in porous media, the capillary forces provide a greater film stability in pore throats. Therefore, reducing the gas diffusion between bubbles of different sizes, which causes coalescence, is an effective way to enhance foam stability in porous media.

The most commonly used gases are N_2_ and CO_2_. CO_2_ can dissolve in crude oil, reducing its viscosity, and the CO_2_ retained in the reservoir can achieve carbon sequestration, which positively contributes to mitigating global climate change [21,22]. However, as the pressure increases, particularly beyond the supercritical pressure, it becomes more challenging to generate strong CO_2_ foam, and the blocking efficiency of the foam rapidly declines [23,24,25]. It is important to note that the solubility of CO_2_ in water and crude oil is approximately 50 times higher than that of N_2_, and this difference in solubility further increases with rising temperature and pressure [26,27]. While the dissolution of a large amount of CO_2_ in crude oil can reduce its viscosity to some extent, its dissolution in water does not significantly improve the mobility of the oil [28]. However, during heavy oil recovery, the average water content in the reservoir is typically as high as 30–50%, and, in the high-permeability channels through which the foam flows, the water content can exceed 70%. Consequently, as CO_2_ diffuses and dissolves in water and crude oil over time, the actual foam quality in the reservoir gradually decreases. Whether foam can be formed and effectively block the reservoir during subsequent flow becomes a critical issue that warrants attention [29].

Additionally, CO_2_ dissolving in water forms carbonic acid (H_2_CO_3_), which increases the acidity of the solution. For example, at 80 °C and 5.07 MPa, the pH of the H_2_O-CO_2_ system can drop to 3.28 [30]. The combination of high temperatures during heavy oil thermal recovery and acidic conditions can cause the hydrolysis of traditional foaming agents such as alkyl ammonium surfactants and sulfate anionic surfactants, leading to a loss of foam stability [31,32].

N_2_, an inert gas widely present in the atmosphere, can be used to partially replace CO_2_ during foam generation as an effective solution to address the issue of foam quality degradation caused by the high solubility of CO_2_ in reservoirs. The literature indicates that N_2_ exists in a subcritical state under most reservoir conditions [33,34]. N_2_ can remain as free gas in the reservoir for extended periods, continuously generating new foam during gas–liquid flow. Additionally, the diffusion coefficient of N_2_ across the liquid film is much lower than that of CO_2_, which helps maintain the stability of the foam in porous media [35].

Betaine surfactants have garnered increasing attention due to their adaptability to high-temperature and high-salinity reservoir conditions [36,37,38,39]. These surfactants are typically derived from natural fatty acids and betaine, but they can also be synthesized chemically. Betaine surfactants are cost-effective and environmentally friendly [40]. They demonstrate good foaming performance in mildly acidic environments created by CO_2_ dissolution in reservoir brines, but they may partially fail in environments with a pH below 4. To enhance the performance of these surfactants in CO_2_ foam, reducing the CO_2_ proportion in the gas phase or incorporating sulfonate groups into the betaine molecules can be effective [25,41]. In addition to chemical modifications of the surfactant structure, another common approach to improve surfactant performance is the formation of composite foams, such as by adding solid nanoparticles [42] or polymers, or blending with other types of surfactants [25].

In steam thermal recovery, it is common to inject mixed gases of nitrogen and carbon dioxide in varying proportions. This study investigates the effect of carbon dioxide concentration in the gas mixture on the bulk stability of a polymer-enhanced betaine surfactant foam system and evaluates the blocking performance of such foam in porous media at the steam front. Currently, there is limited research on the stability of foam in the high-temperature region of the steam front. The findings of this study are highly valuable for reference.

## 2. Materials and Methods

### 2.1. Material

Cocamidopropyl hydroxysultaine (CHSB) is a high-temperature, high-salinity-resistant surfactant, synthesized by bridging cocamide with betaine via a propyl group. This surfactant exhibits zwitterionic characteristics, containing ammonium ions, carboxyl groups, and sulfonate groups within its molecular structure [43]. CHSB has demonstrated excellent thermal and salt tolerance in both laboratory evaluations and field applications, and it is supplied by Green Forest Chemical Company (Linyi, China). Additionally, a highly temperature-resistant polymer, designated as Z364, was selected for this study, provided by the Sinopec Exploration and Production Research Institute. The Z364 polymer features a thermally stable long-chain saturated alkyl backbone –(CH_2_–CH_2_)ₙ– and is synthesized through the polymerization of N-methylol butenone carboxylate, p-vinylbenzene sulfonate, and pyrrolidone monomers. Z364 possesses resistance to high temperatures, salinity, and acidity, with a molecular weight of up to 10 × 10^6^ g/mol, making it suitable for enhancing the stability of CHSB foam at the steam front. The polymer-enhanced foam (PEF) formulated with these two chemical agents was used to study the effect of CO_2_ concentration on the plugging performance in high-permeability channels at the steam front. The simulated formation water had a salinity of 50,000 mg/L, with NaCl content at 42.85 g/L and CaCl_2_ content at 7.15 g/L. The concentration of CO₂ and N₂ is 99.9% (Tianyuan Gas Co., Qingdao, China)., were used to generate the foam.

### 2.2. High-Temperature High-Pressure Foam Experiment

**Figure 1:**
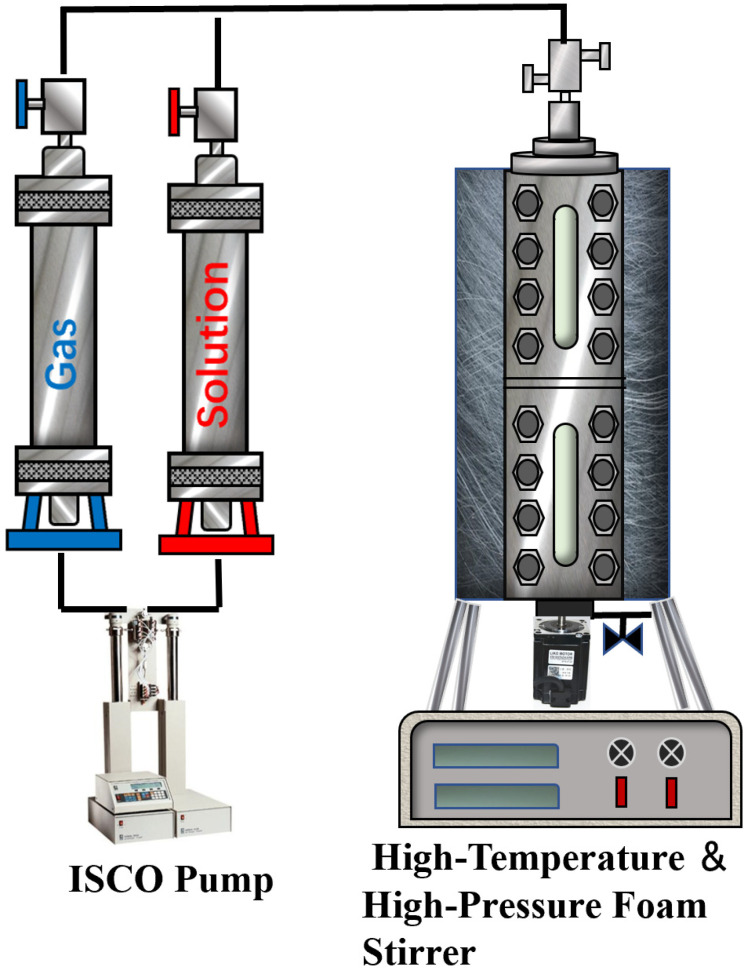
High-temperature high-pressure foam testing apparatus.

The high-temperature and high-pressure foam meter is a pressure-resistant vessel equipped with a viewing window and graduated scale. At the start of each experiment, 100 mL of prepared foaming solution is added to the foam meter. After evacuating the stirrer, the prepared gas is injected, and the temperature and pressure of the high-temperature, high-pressure chamber are adjusted. Considering the significant difference in solubility between CO_2_ and N_2_ in the solution, to minimize the impact of mass transfer in a non-equilibrium state on the experimental results, the gas and solution are stirred at a low speed for more than 0.5 h until the temperature and pressure stabilize, forming a balanced fluid. Subsequently, the balanced fluid is stirred at a high speed of 1000 r/min for 10 min. The foam volume (V) is obtained through the viewing window, and the time required for the foam volume to reduce by half (T_1_/_2_) is recorded to evaluate foam stability. Each set of samples is measured three times, and the average value of the experimental data is taken. The experimental procedure is shown in Figure 1.

### 2.3. Interfacial Tension

An interfacial rheometer (Tracker-H) was used to measure the viscoelastic modulus and interfacial tension of the surfactant solution. The viscoelastic modulus is an indicator of the foam film’s ability to resist elastic deformation. First, the prepared gas was injected into the high-pressure chamber, and the temperature and pressure within the chamber were adjusted accordingly. A pendant drop of water in the shape of a pear was then created using the high-pressure chamber and syringe, and the droplet’s profile was recorded using a CCD camera. The interfacial tension was calculated using the Gauss–Laplace equation, and the droplet profile was analyzed by the accompanying software Windrop 3.2.9.

### 2.4. Measurement of Solution Rheology

The rheological properties of the solution and foam were measured using a modular rheometer (Anton Paar MCR302, Anton Paar GmbH, Graz, Austria). The experiment was conducted at room temperature. During rotational measurements, the rheometer’s rotor rotates at a shear rate of 7.34 s^−1^, while the outer cylinder remains stationary. The rotating rotor induces laminar flow in the liquid within the annular space, applying strain or stress through continuous rotation, and the corresponding torque values are measured. In rotational measurements, the shear plane is defined by the concentric cylindrical surfaces, with the shear line corresponding to a circle on the shear plane perpendicular to the axis. The motion of the fluid elements coincides with the shear line. The shear stress and shear rate are calculated based on the measured torque using the following formula:

(1) $$\tau =\frac{M}{2\pi R_{1}^{2}\left(R_{a}-R_{1}\right)}$$

(2) $$\gamma^{\prime }=\frac{R_{1}n}{\left(R_{a}-R_{1}\right)}$$

In the formula, *M* represents the torque, *R*_1_ = 25 mm is the radius of the rotor, and *R_a_* = 27 mm is the radius of the cylinder. *n* is the rotational speed of the cylindrical rotor, with the unit being revolutions per minute (rpm). The viscosity of the fluid at different shear rates is determined by solving the constitutive equation of the fluid.

### 2.5. Foam Flow Experiment in Core

**Figure 2:**
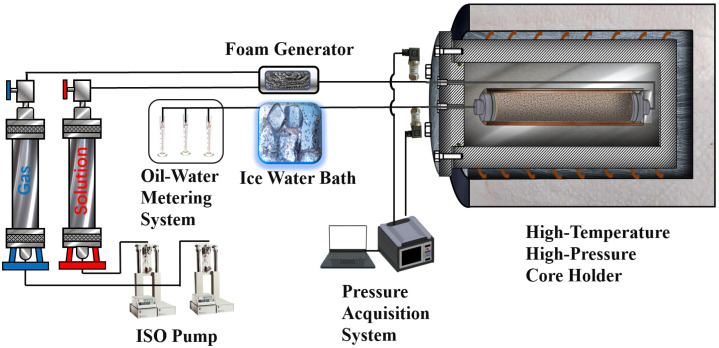
High-temperature high-pressure core experiment flowchart.

The Berea core was placed inside a copper sleeve within the core holder, with pipelines connected at both ends. The copper sleeve was positioned in the high-temperature, high-pressure chamber, and a confining pressure of 10–20 MPa was applied to the annulus of the chamber to ensure the copper sleeve was securely pressed against the core surface. The high-temperature, high-pressure chamber was then placed in an electric heating furnace, where the temperature and confining pressure of the apparatus were adjusted. The back pressure was set higher than the steam saturation pressure at the current temperature. A pre-determined volume (1 PV) of surfactant solution was injected into the core to achieve saturation adsorption on the core surface. The pressure differential across the sand-packed tube, Δp1, was recorded after the solution injection. Subsequently, both the surfactant solution and high-pressure gas were simultaneously injected into the core, with the solution injection rate set at 1 mL/min and the foam mass fraction at 60%. The pressure differential across the core, Δp2, was recorded. The experimental procedure is illustrated in Figure 2. The foam resistance factor can be calculated using Equation (3):

(3) $$Z=\frac{\Delta p_{2}}{\Delta p_{1}}$$

## 3. Results and Discussion

### 3.1. Effect of Carbon Dioxide Concentration in Gas on Foam Performance

**Figure 3:**
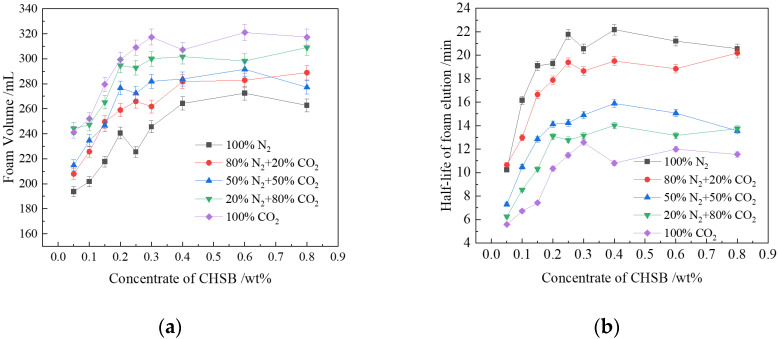
Effect of CO_2_ concentration in gas on CHSB foam: (**a**) foam volume; (**b**) foam half-life (2 MPa, 100 °C, salinity: 50,000 mg/L).

Figure 3a illustrates the foaming performance of the CHSB surfactant in gases with varying CO_2_ concentrations. As the concentration of the surfactant increases, the adsorption of its molecules at the gas-liquid interface also increases, which reduces the free energy required for foam generation, leading to an increase in foam volume and foam half-life [44]. However, when the surfactant concentration exceeds the critical micelle concentration (CMC), the adsorption concentration of the surfactant at the gas–liquid interface decreases, and more surfactant molecules form micelles within the liquid phase. This reduces the rate at which surfactant molecules replenish the gas-liquid interface, thereby weakening the ability to maintain the interface layer thickness via the Gibbs-Marangoni effect [1,45]. Consequently, when the surfactant concentration exceeds 0.3%, both the foam volume and half-life exhibit fluctuations at higher levels and slight decreases. Thus, in subsequent experiments, the surfactant concentration was fixed at 0.3%.

The experimental results indicate significant differences in the foaming ability and foam stability of the surfactant solution in gases with different CO_2_ concentrations. The volume of pure CO_2_ foam ranges from 241 to 317 mL, while the volume of pure N_2_ foam ranges from 193 to 262 mL, showing a decrease of approximately 18–20% in N_2_ foam volume compared to CO_2_ foam. In a pure CO_2_ gas environment, the surfactant’s foaming ability is superior to that under pure N_2_ conditions [46]. When the gas is a mixture, the foaming ability of the surfactant increases with CO_2_ concentration. Subsequent experiments confirmed that this phenomenon is related to the gas–liquid interfacial tension [47]. The interfacial tension between CO_2_ and water is much lower than that between N_2_ and water [48,49], as a lower interfacial tension requires less energy for foam generation [50].

Figure 3b shows a strong correlation between the foam drainage half-life (T_1/2_) and the CO_2_ concentration in the gas phase. The volume half-life (T_1/2_) of N_2_ foam is approximately 2.6 times that of CO_2_ foam. Increasing the proportion of N_2_ in the gas mixture enhances foam stability. When the N_2_ proportion is increased from 50% to 80%, the foam volume half-life (T_1/2_) improves by 40% to 70%. The foam half-life is primarily related to the rupture of the foam liquid film due to liquid drainage and the coalescence of bubbles caused by gas diffusion. After foam generation, gravity causes the liquid within the film to gather at the bottom, thinning and weakening the upper foam layer, which accelerates foam rupture [12,51]. As liquid drains from the film, a surface tension gradient is created. The Gibbs-Marangoni effect generates a counteracting force, causing a small amount of liquid to flow back into the dry foam, repairing and stabilizing the film. This process occurs before the film reaches a critical thickness that leads to rupture [14]. As the surfactant concentration increases, more surfactant molecules adsorb at the gas-liquid interface, thickening the liquid film and slowing the liquid drainage process.

When the differences in liquid film thickness and the permeability coefficient of the surfactant monolayer are small, the liquid film permeability coefficient is mainly influenced by the gas diffusion coefficient in the liquid phase and the gas solubility in the liquid [52]. The mass transfer rate of gas through the foam liquid film can be characterized by the film permeability defined by the Princen and Mason equation [53]:

(4) $$K=\frac{D_{w}\cdot H}{h_{2}+2D_{w}/k_{m1}}$$

In the equation, K represents the liquid film permeability coefficient, with units of cm·s⁻^1^; *D_w_* is the diffusion coefficient of the gas in the liquid phase of the liquid film middle layer, with units of cm^2^·s^−1^; *H* is the Ostwald coefficient representing the solubility of the gas in the liquid phase; *h*_2_ is the thickness of the liquid core in the middle layer of the liquid film, with units of cm; and *k_m_*_1_ is the permeability coefficient of the surfactant monolayer, with units of cm·s^−1^.

From Equation (4), it can be seen that the liquid film thickness, surfactant monolayer permeability, gas diffusion coefficient in the liquid phase of the middle layer, and gas solubility in the solution are the main factors affecting the permeability of the foam liquid film [54]. When there are minimal differences in liquid film thickness and surfactant monolayer permeability, the liquid film permeability coefficient is primarily influenced by the gas diffusion coefficient in the liquid phase and its solubility [55]. At 25 °C in pure water, the K values for CO_2_ and N_2_ are 2.937 × 10^6^ and 155 × 10^6^ Pa·L·mol^−1^, respectively. The diffusion coefficients of the two gases in the liquid phase are not significantly different, which is why the liquid film permeability coefficient for CO_2_ foam is much higher than that for N_2_ foam, explaining why CO_2_ foam is less stable than N_2_ foam [47,56].

The relationship between the liquid film permeability KG of a multi-component gas and its components can be expressed by the following equation [47]:

(5) $$K_{G}={\left(\Sigma_{i=1}^{n}\frac{x_{i}}{K_{i}}\right)}^{-1}$$

In the equation, *n* represents the number of components, *x_i_* is the mole fraction of component I, and *K_i_* is the permeability of the liquid film to the gas component i. In a multi-component gas system, the gases diffuse independently within the liquid film, and the rate at which a gas passes through the liquid film is related to its mole fraction within the bubble. The addition of N_2_ reduces the mole fraction of CO_2_, and, although the permeability of the liquid film to CO_2_ does not decrease, the diffusion rate of CO_2_ through the liquid film slows significantly as its mole concentration in the bubble decreases [36]. On a macroscopic scale, the introduction of a certain concentration of N_2_ effectively reduces the rate of bubble coalescence [57]. As the relative concentration of N_2_ increases, the diffusion rate of CO_2_ in the mixed gas decreases significantly. The higher the concentration of CO_2_ in the gas phase, the faster the average diffusion rate, leading to a shorter foam volume half-life (T_1/2_).

### 3.2. The Effect of Polymer on Foam Stability

**Figure 4:**
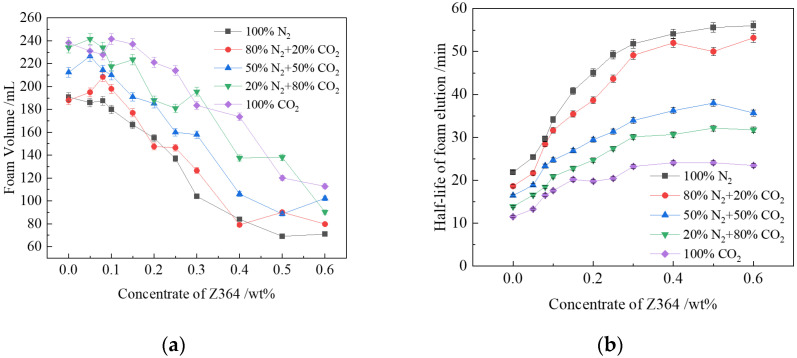
Relationship between CO_2_ concentration in gas and the volume and half-life of polymer-enhanced foam at different polymer concentrations: (**a**) foam volume; (**b**) foam half-life (2 MPa, 100 °C, 0.4% CHSB concentration, salinity: 50,000 mg/L).

The addition of the Z364 polymer reduced the foaming volume of CHSB foam. When the polymer concentration exceeded 0.2%, the foam volume decreased significantly. The increase in solution viscosity due to the polymer addition required more energy to disperse the gas phase into the liquid phase to generate foam, but the stirring speed of the experimental apparatus was relatively low, providing limited energy [19,20,41]. As shown in Figure 4a, the higher the CO_2_ concentration in the gas, the larger the foam volume. The volume of pure CO_2_ foam is 30–35% greater than that of pure N_2_ foam. As the polymer concentration increases, the foam volume produced by various gases decreases, but the decrease is less pronounced for gases with higher CO_2_ concentrations. With higher polymer concentrations, the gas-water interfacial tension of CO_2_ decreases, making foam generation easier. During the preparation of the experiment, CO_2_ dissolves fully in the solution, and foam forms when CO_2_ is released during stirring [25,58]. The foam formed in this process becomes more stable as the solution viscosity increases.

Figure 4b shows that the addition of the polymer significantly improves the foam stability. Adding just 0.1% polymer can increase the foam volume half-life by 47–63%. The reduction in CO_2_ concentration in the gas phase enhances foam stability more markedly. This is primarily because the addition of Z364 polymer increases the viscosity of the foam liquid film, slowing the drainage process [59]. The liquid drainage rate in the foam film can be described by the following equation [51,60]:

(6) $$v_{RE}=-\frac{dh_{f}}{dt}=\frac{2h_{f}^{3}}{3\eta_{c}r_{film}^{2}}\Delta p_{film}$$

In the equation, *V_RE_* represents the liquid film drainage rate, measured in m/s; *h_f_* is the liquid film thickness, in meters; t is the time, in seconds; *r_film_* is the radius of curvature of the liquid film, in meters; *η_c_* is the viscosity of the solution, in Pa·s; and Δ*p_film_* is the pressure difference across the liquid film, in Pa.

The increase in liquid film thickness is related to the formation of micelle structures and network structures by polymer molecules within the liquid film, which enhances the film’s viscoelasticity. This reduces the likelihood of film rupture when subjected to disturbances [54]. The difficulty for gas molecules to pass through the adsorption layer and the liquid film layer increases, creating a higher energy barrier at the interface, making it more difficult for gas molecules to pass through the gaps between surfactant molecules. As a result, the bubble disproportionation effect caused by gas diffusion is delayed, significantly improving the foam stability. According to Equation (5), the lower the CO_2_ concentration in the gas phase, the more pronounced the polymer’s inhibitory effect on gas diffusion. The experimental results also indicate that the polymer can extend the stability of N_2_ foam by 35 min, but, as the CO_2_ concentration increases, the extension of the stability time gradually decreases to 12 min.

Another noteworthy phenomenon is that adding 20% CO_2_ to N_2_ results in only about a 10% reduction in foam stability. In contrast, adding 20% N_2_ to CO_2_ can increase foam stability by approximately 50%. This suggests that, during the disproportionation process in high-concentration CO_2_ foam, when high-concentration CO_2_ in small bubbles diffuses through the liquid film into larger bubbles, the remaining N_2_ concentration gradually increases. Due to the slower diffusion process of N_2_, the presence of small bubbles is effectively prolonged. The addition of the polymer improves the liquid film stability, reducing the probability of bubble coalescence due to film rupture. Therefore, the presence of N_2_ extends the lifespan of small bubbles. In summary, under the influence of the polymer, the stabilizing effect of N_2_ on foam in high-concentration CO_2_ gas is amplified [27,48,61].

### 3.3. Thermal Stability of Foam

**Figure 5:**
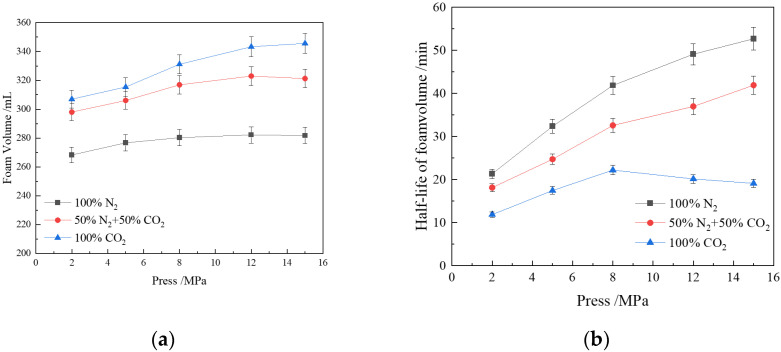
Effect of CO_2_ concentration in gas on CHSB mixed foam under different pressures: (**a**) foam volume; (**b**) foam volume half-life (2 MPa, 100 °C, 0.4% CHSB concentration, salinity: 50,000 mg/L).

The effects of temperature on the two types of foam are shown in Figure 5. Although the CHSB surfactant has a relatively good thermal stability, increasing temperatures still negatively impact foam stability. As shown in Figure 5a, within the temperature range of up to 50 °C, the increase in temperature reduces the viscosity of the solution, and enhances the migration speed of surfactant molecules at the gas–liquid interface, thereby lowering the interfacial tension and increasing foam volume. As a result, the solution exhibits an improved foaming ability. However, as the temperature continues to rise, more surfactant molecules form micelles, leading to a decrease in the number of molecules adsorbed at the gas-liquid interface. This reduces the surface tension gradient, weakening the strength and self-repair ability of the liquid film, causing the foam volume to begin declining [20,24]. The rise in temperature not only destabilizes the liquid film and increases the probability of film rupture but also accelerates the thermal motion of gas molecules, increasing the coalescence rate between bubbles of different sizes [33,41]. The drainage half-life of CHSB foam decreases significantly, with the foam half-life at 90 °C being reduced by 54.7% compared to that at 30 °C.

Figure 5a illustrates the relationship between foam volume and pressure. Within the pressure range of 2–15 Mpa, the foam volume increases with rising gas pressure, with this effect being more pronounced at lower pressures. As the density of gas molecules within the bubbles increases, the compressive effect on the surfactant molecules at the gas–liquid interface intensifies [50]. The polar parts of CHSB surfactant molecules tend to align towards the liquid phase, while the hydrophobic parts orient towards the gas phase. Under high pressure, this arrangement becomes more compact, reducing the interfacial free energy and consequently lowering the surface tension, ultimately leading to an increase in foam volume [62].

There are significant differences in the foaming volume among the three gases, with the increase in foam volume due to pressure being less pronounced for N_2_ compared to the other two gases. In a CO_2_ environment, CHSB surfactant molecules can more effectively form a stable interfacial film at the gas-liquid interface, encapsulating more gas [36,63]. This reduction in surface tension results in a larger foam volume, as more gas can be stabilized within the foam structure. Additionally, the solubility of CO_2_ in the solution increases under high pressure [64]. During foam generation, CO_2_ precipitates from the solution, forming a large number of small bubbles, which further increases the foam volume. When the CO_2_ concentration in the gas phase is 50%, the foam volume is closer to that of pure CO_2_ foam.

The impact of pressure on the foam volume half-life is also significant, as shown in Figure 5b. As the pressure increases, the gas density rises, reducing the density difference between the gas and liquid phases, which slows down the liquid drainage from the film. With the increase in CO_2_ density, the angle between the head and tail vectors of the surfactant molecules relative to the interface normal decreases, facilitating the embedding of the hydrocarbon end of the CHSB surfactant molecules into the CO_2_ phase [58]. The arrangement of surfactant molecules becomes tighter, increasing the thickness of the liquid film, and thus enhancing the stability of CO_2_ foam with increasing pressure [65,66]. However, when the pressure reaches a critical state, the extraction of CHSB surfactant molecules by CO_2_ intensifies, disrupting the stable adsorption of surfactants at the gas–liquid interface [67]. Simultaneously, the permeability of CO_2_ between adjacent bubbles also increases with pressure, leading to reduced foam stability. The increase in pressure also enhances the attraction of N_2_ to the hydrophobic ends of surfactant molecules. Combined with the lower solubility and chemical stability of N_2_ in the solution, this results in a more significant improvement in foam stability. When 50% of the CO_2_ mole fraction is replaced by N_2_, the lower permeability of N_2_ in the liquid film allows the mixed gas to effectively maintain bubble integrity, extending the foam volume half-life.

### 3.4. Effect of Temperature on Foam Performance

**Figure 6:**
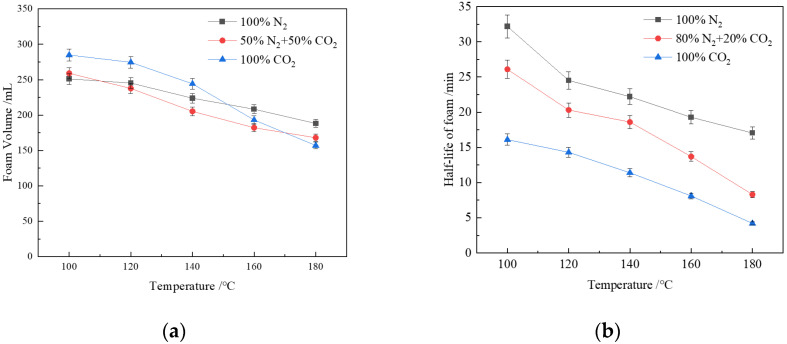
Temperature resistance of CHSB foam with different gases: (**a**) foam volume; (**b**) foam volume half-life (6 MPa, 0.4% CHSB concentration, salinity: 50,000 mg/L).

To prevent the solution from boiling under high-temperature conditions, the pressure of the high-temperature high-pressure foam testing apparatus was set to 6 Mpa, where the critical temperature of the steam is 275 °C. The foam volume and its half-life (T_1/2_) for the CHSB solution mixed with different gases are shown in Figure 6. CHSB was still able to effectively generate foam at temperatures as high as 180 °C. However, when the temperature exceeded 100 °C, the stability of surfactant molecules at the gas–water interface was negatively impacted. The foam volume of N_2_ decreased by 25.1% with increasing temperature, while the foam volume of CO_2_ decreased by 44.9%, indicating that CO_2_ has a weaker gas–liquid interface stability. When the temperature exceeded 150 °C, an increase in CO_2_ concentration led to a reduction in foam volume. Replacing 50% of the CO_2_ concentration with N_2_ resulted in a greater temperature effect on the foam volume of the mixed gas, suggesting that even low concentrations of CO_2_ can significantly negatively affect the foaming ability of the solution under high-temperature conditions [15,68].

The negative impact of increasing temperature on foam volume half-life intensifies with increasing CO_2_ concentration in the gas phase. This is mainly due to several factors: First, as the temperature rises, water evaporation from the foam liquid film accelerates, causing the film to thin and the drainage rate to increase, making the foam more prone to rupture. Second, the increase in temperature reduces the viscosity of the surfactant solution, which accelerates the liquid drainage rate from the film, leading to decreased foam stability [69]. Additionally, the increase in temperature affects the mass transfer, adsorption, and desorption at the gas-liquid interface, as well as molecular thermal motion behaviors and physical processes. According to the gas kinetic theory, as the temperature rises, the average energy of gas molecules increases, leading to a higher collision frequency between gas molecules and surfactant molecules at the interface, accelerating the Ostwald ripening process of the bubbles [70]. The stability of N_2_ foam is higher than that of CO_2_ foam, which is related to the lower solubility of N_2_ in the solution compared to CO_2_, and the much lower permeability coefficient of N_2_ molecules through the liquid film. When the temperature does not exceed 150 °C, the behavior of the mixed gas foam is closer to that of N_2_ foam, but, at higher temperatures, the disruption of liquid film stability by CO_2_ becomes the main cause of foam collapse and coalescence [71].

### 3.5. Interfacial Tension of the Solution

#### 3.5.1. Effect of CO_2_ Proportion in Gas on Gas–Water Interfacial Tension

**Figure 7:**
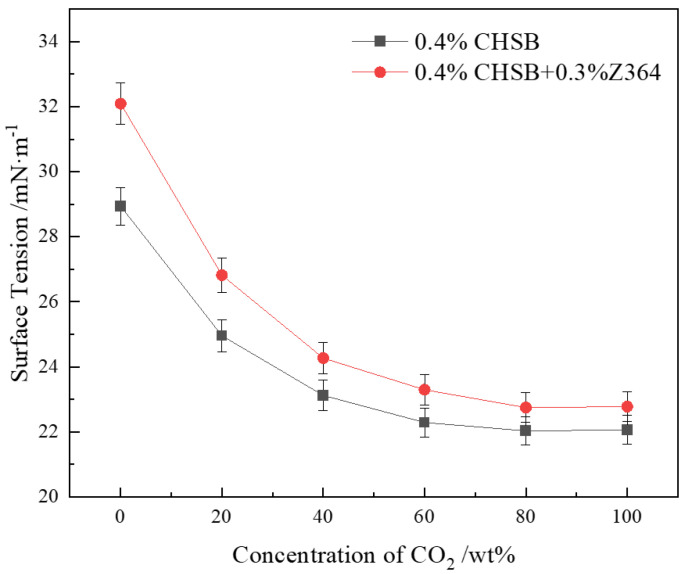
Effect of CO_2_ concentration in N_2_ and CO_2_ mixed gas on interfacial tension (2 MPa, 100 °C).

CO_2_ has a higher solubility in the solution compared to N_2_. As the concentration of CO_2_ increases, more CO_2_ molecules adsorb at the interface between the surfactant and the gas, leading to a reduction in interfacial tension [72]. As shown in Figure 7, the interfacial tension tends to decrease with increasing CO_2_ concentration. The mixed gas of N_2_ and CO_2_ exhibits certain non-ideal behavior, where their effects at the interface do not simply add up proportionally. The CO_2_ molecules at the gas–liquid interface gradually approach saturation [50]. Once the interface approaches saturation, further increases in CO_2_ concentration have a diminishing effect on interfacial tension, causing the changes in tension to level off [70].

The addition of the Z364 polymer increases the interfacial tension at the gas–liquid interface. The presence of ionic groups in the Z364 polymer imparts upon it some interfacial adsorption capability, partially replacing the adsorption sites of CHSB surfactant molecules at the gas-liquid interface [73,74]. However, due to the larger molecular weight and longer molecular chains of the Z364 polymer, its ability to reduce interfacial tension is weaker than that of the surfactant. CO_2_, with its higher solubility and stronger polarity, exerts a stronger attraction with water molecules, thereby diminishing the polymer’s effect on reducing interfacial tension.

#### 3.5.2. Effect of Pressure on Gas-Liquid Interfacial Tension

**Figure 8:**
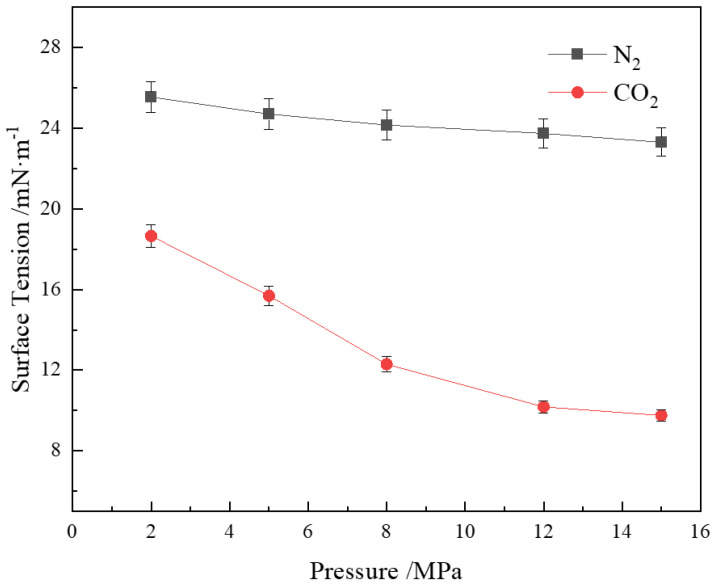
Effect of pressure on interfacial tension (100 °C, 0.4% CHSB concentration).

Figure 8 shows that the interfacial tension of the CHSB solution in CO_2_ and N_2_ gases decreases with increasing pressure. According to Henry’s law, the solubility of a gas is directly proportional to pressure [8]. Due to the non-polar nature and low solubility of N_2_, its dissolution in water is relatively limited, making the reduction in interfacial tension less pronounced as pressure increases. In contrast, the solubility of CO_2_ in water increases significantly under pressure. This high solubility allows CO_2_ molecules to form a dense adsorption layer at the gas–liquid interface, markedly reducing the interfacial free energy and, consequently, the interfacial tension [70]. Under high pressure, the dissolved CO_2_ molecules can more effectively rearrange and enhance interface adsorption, further reducing the interfacial tension. Additionally, the dissolution of CO_2_ under high pressure increases the acidity of the solution, leading to the protonation of the carboxylic acid groups in the CHSB surfactant [12,49,74]. This protonation effect reduces the polarity and intermolecular electrostatic repulsion of CHSB molecules, allowing more surfactant molecules to adsorb at the gas–liquid interface, thereby further decreasing the interfacial tension.

#### 3.5.3. Effect of Temperature on Interfacial Tension

**Figure 9:**
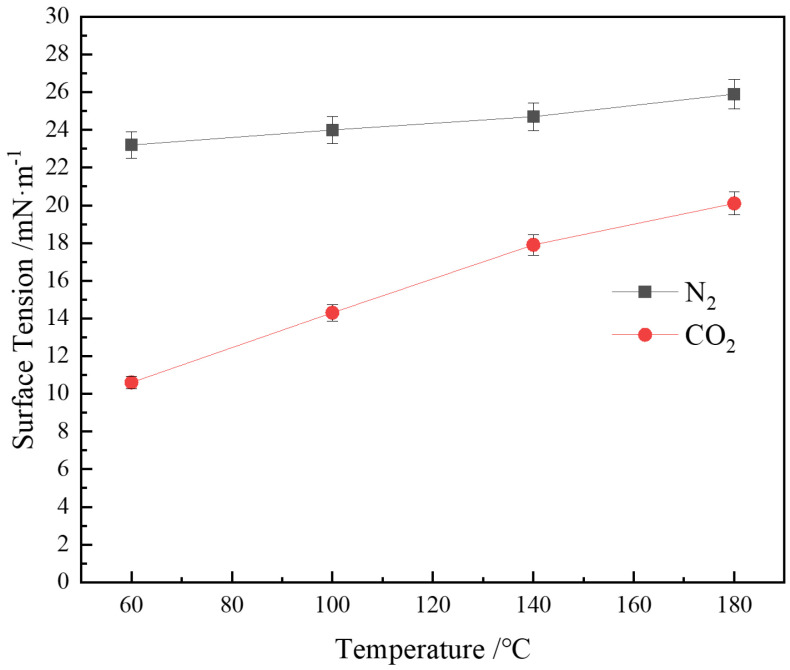
Effect of temperature on interfacial tension (6 MPa, 0.4% CHSB concentration).

Figure 9 shows that the increase in temperature has a more significant impact on the interfacial tension between CO_2_ and the CHSB solution. This is primarily because the solubility of CO_2_ decreases significantly with rising temperature, reducing the adsorption of CO_2_ at the gas-liquid interface [15]. The reduction in CO_2_ solubility decreases the concentration of carbonate ions in the solution, leading to a lower degree of protonation of the carboxylic acid groups in the CHSB surfactant. This increases the hydrophilicity of the surfactant molecules, thereby reducing their adsorption capacity at the interface [75]. In contrast, N_2_ molecules, being more inert and having a low solubility in water, show a weaker temperature dependence in their adsorption behavior at the gas-liquid interface. Consequently, even as the temperature rises, the arrangement and adsorption characteristics of N_2_ molecules at the interface change little, resulting in only minor variations in interfacial tension [61].

Additionally, the increase in temperature raises the kinetic energy of CHSB molecules, making them more likely to detach from the interface into the water phase or rearrange, leading to fluctuations in surfactant density and the formation of voids in the gas–liquid film. These voids are formed by spatial fluctuations (interface waves or dimples) and surfactant density fluctuations within the gas–liquid film [76]. Such fluctuations enlarge the holes on the gas–liquid interface, accelerating the drainage from the film and gas transfer between bubbles [65]. On a macroscopic scale, bulk foam exhibits an increase in gas–liquid interfacial tension with rising temperature, resulting in decreased foam stability [62].

### 3.6. Rheology of Solution and Foam

**Figure 10:**
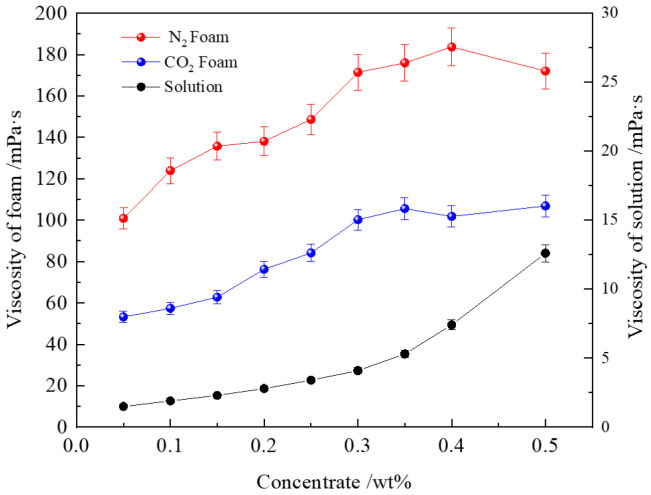
Viscosity of PEF and PEF solution (0.4% CHSB concentration).

Figure 10 shows the viscosity curve of the PEF solution. When the polymer concentration is low, the viscosity increases slowly. At this stage, the Z364 polymer molecules are relatively sparse in the solution, with minimal physical cross-linking or entanglement between molecules. The viscosity of the solution is mainly determined by the mobility of individual polymer molecules [27,77]. As the concentration increases, the viscosity shows a slow, approximately linear growth. However, as the Z364 polymer concentration further increases, the interactions between molecules intensify, leading to the formation of more complex entanglements and network structures. This significantly restricts the fluidity of the solution, causing the viscosity to increase exponentially, with a marked nonlinear rise in viscosity [18].

The shear viscosity of polymer-enhanced foam also increases significantly with the polymer concentration. Low concentrations of the polymer not only enhance the viscosity of the liquid film but also substantially improve the viscoelasticity of the gas-liquid interface. On a macroscopic level, this manifests as a more pronounced increase in foam viscosity under shear stress compared to the polymer-surfactant solution alone. Figure 10 shows that the viscosity of N_2_ foam is higher than that of CO_2_ foam, which can be attributed to the higher solubility of CO_2_ in the surfactant solution [77], while N_2_ foam exhibits a greater stability and higher liquid film viscoelasticity [61]. In porous media, the combination of low solution viscosity and high apparent foam viscosity helps improve the effectiveness of foam in blocking high-permeability channels. Reducing the proportion of CO_2_ in CO_2_ foam and adding an appropriate amount of N_2_ can enhance the potential application of foam in enhanced oil recovery (EOR) practices.

**Figure 11:**
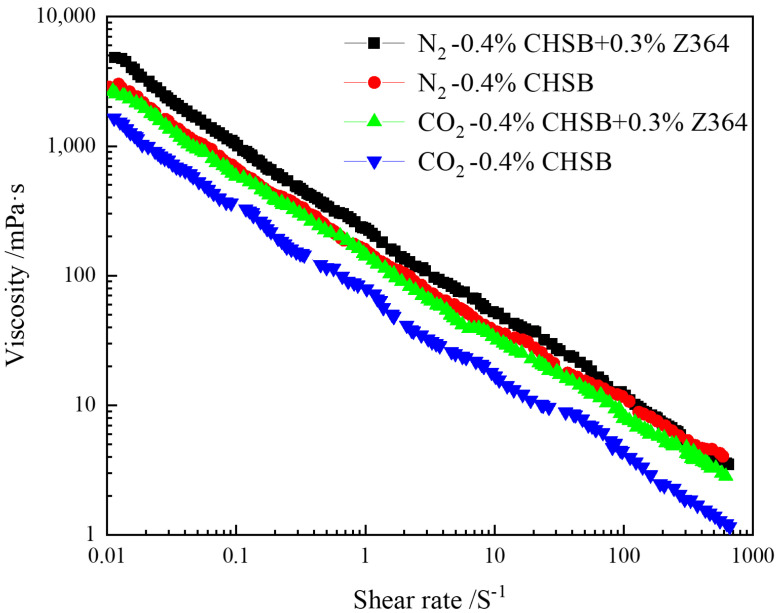
Variation of polymer viscosity with shear rate.

When foam fluid is injected into the reservoir, the varying sizes of pores, throats, and walls exert compressive, collisional, and shearing forces on the foam, leading to partial foam rupture. This results in a reduction in foam viscosity, thereby weakening its plugging ability [61]. Therefore, the strength of the foam is crucial in determining its effectiveness in providing a sealing function [34]. The rheological properties of different foam systems were tested using a rheometer, and the results are shown in Figure 11. As the shear rate increases, the apparent viscosity of the foam profile control system decreases continuously. The foam model conforms to the power-law model. This indicates that the slower the flow rate of the foam, the greater its apparent viscosity. The addition of the polymer enables the foam system to generate a greater flow resistance, which is reflected macroscopically as a higher viscosity and enhanced stability.

### 3.7. Study on the Plugging Effect of Foam in Core Samples

The stability of foam systems is commonly studied using bulk foam methods in laboratory settings. However, the behavior of foam systems in porous media can differ significantly from that of bulk foam. To more accurately reflect the flow control effect of foam at the steam front, a series of core flow experiments were conducted. The parameters for the core experiments are shown in Table 1. Different foam systems were injected into Berea sandstone cores with a permeability of approximately 490–530 mD to study their plugging effectiveness at the steam front. Unless otherwise specified, the polymer-enhanced foam (PEF) solution was composed of 0.4% CHSB surfactant and 0.3% Z364 polymer.

#### 3.7.1. Effect of CO_2_ Concentration in Gas

**Figure 12:**
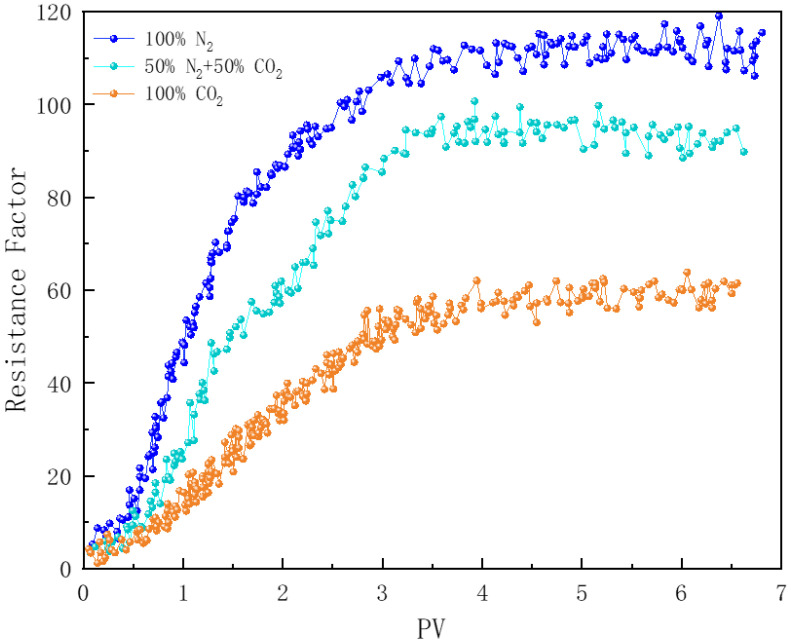
Effect of CO_2_ concentration in gas on foam plugging strength (150 °C).

In Figure 12, core samples numbered 2, 3, and 4 demonstrate the effect of CO_2_ concentration in the gas phase on foam plugging performance. Under a back pressure of 6 MPa, the resistance factor of N_2_ foam reaches 115, which is 2.1 times higher than that of pure CO_2_ foam. Previous studies have shown that N_2_ foam exhibits a greater stability compared to CO_2_ foam; therefore, in porous media, N_2_ foam can more effectively penetrate deeper into the core and enhance the flow resistance to a greater extent [78]. Additional research indicates that, when the foam quality is below 90%, the flow resistance of the foam increases with rising foam quality [26]. Under identical injected foam quality conditions, due to the lower solubility of N_2_ in water, N_2_ foam maintains a higher foam quality during flow compared to CO_2_ foam. When the proportion of CO_2_ gas is reduced to 50%, the resistance factor curve of the foam closely resembles that of pure N_2_ foam. Polymer-enhanced foam (PEF) effectively counteracts the negative impact of CO_2_ dissolution on liquid film stability. The addition of N_2_ not only improves foam stability but also increases the actual gas proportion—i.e., foam quality—within the flowing foam in the core samples.

#### 3.7.2. Effect of Temperature

**Figure 13:**
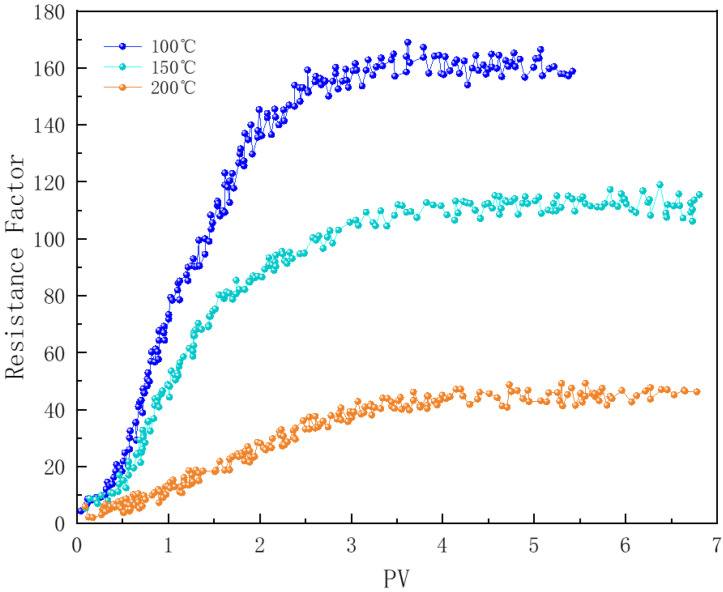
Effect of temperature on foam plugging strength.

In Figure 13, core samples numbered 1, 2, and 6 illustrate the impact of temperature on foam flow within the cores. Temperature is one of the most critical factors affecting the structural stability of surfactants and their adsorption characteristics at the gas-liquid interface [37]. As the fluid temperature in the reservoir increases, particularly near the edges of the steam chamber, the effectiveness of foam plugging varies. The polymer-enhanced foam (PEF) exhibits good plugging performance at 100 °C, with a resistance factor reaching 150–160. However, as the temperature rises to 150 °C, the strength of the liquid film decreases, causing the resistance factor to drop to around 110. When the temperature further increases to 200 °C, the stability of the PEF foam is significantly compromised, with the resistance factor falling below 40. Excessive temperatures weaken both the activity of the surfactant and the viscosity-enhancing effect of the polymer, making bubbles more prone to rupturing and coalescing during flow [37]. At 200 °C, stable foam flow is only achieved after approximately 4 pore volumes (PVs) of foam fluid have been injected.

#### 3.7.3. Effect of Polymer

**Figure 14:**
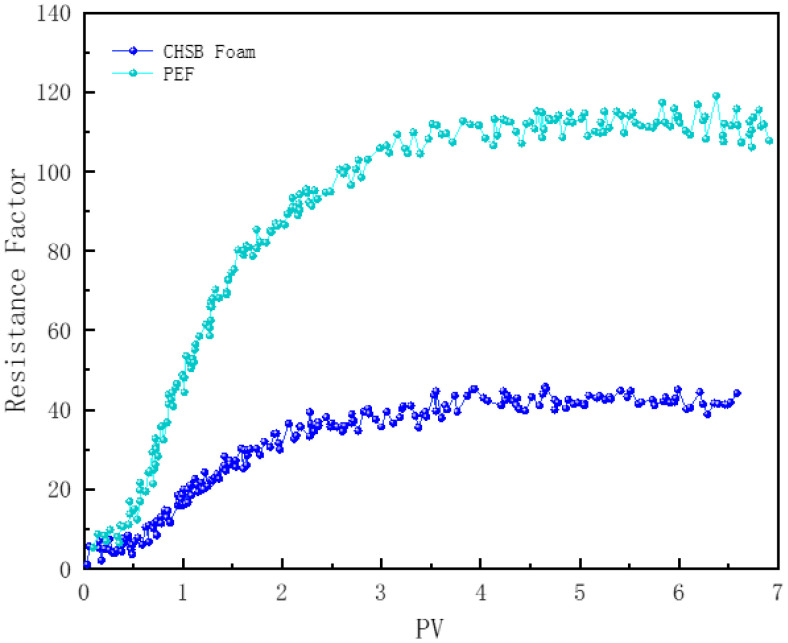
Resistance factor of foam vs. injected foam pore volume (PV).

In Figure 14, core samples numbered 2 and 5 demonstrate the effect of the polymer on foam flow within the cores. The improvement in CHSB foam flow due to the polymer can be attributed to two main factors: first, the polymer increases the viscosity of the solution, thereby enhancing the baseline viscosity of the foam fluid as it flows through the core; second, the polymer strengthens the stability of the liquid film, reducing the likelihood of film rupture when the foam is stretched and compressed as it passes through pore throats [12,26,41]. The gas is effectively separated by the thicker liquid film, significantly increasing the flow resistance of the foam. The flow resistance of polymer-enhanced foam is 2.8 times higher than that of CHSB foam.

#### 3.7.4. Effect of Pressure

**Figure 15:**
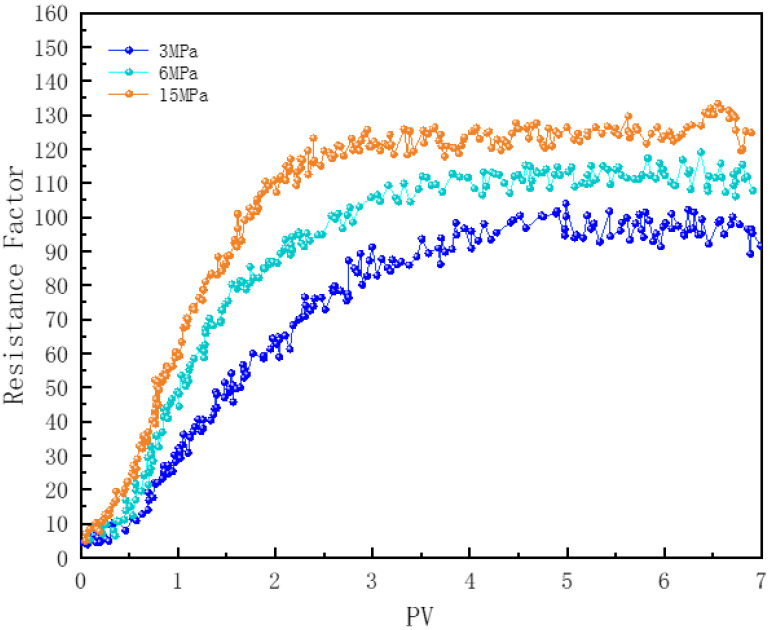
Effect of pressure on foam plugging strength.

In Figure 15, core samples numbered 2, 7, and 8 illustrate the impact of pressure on the flow of N_2_ foam within the cores. Pressure primarily affects the state of the gas and the gas-liquid interfacial tension, thereby influencing the foam’s ability to flow through porous media. Since N_2_ is an inert gas, its dissolution has a minimal effect on the adsorption of chemicals at the gas–liquid interface [78]. As the pressure increases, the density of N_2_ also increases, which reduces the Ostwald ripening effect between bubbles [66]. Unlike the significant enhancement in bulk foam stability with increasing pressure, the effect of pressure on improving the flow resistance of N_2_ foam in porous media is relatively minor [49]. Although the resistance values are very similar after injecting three pore volumes of foam, the time required for the foam to reach equilibrium in porous media is shorter under high pressure [8].

#### 3.7.5. Effect of Gas Solubility

**Figure 16:**
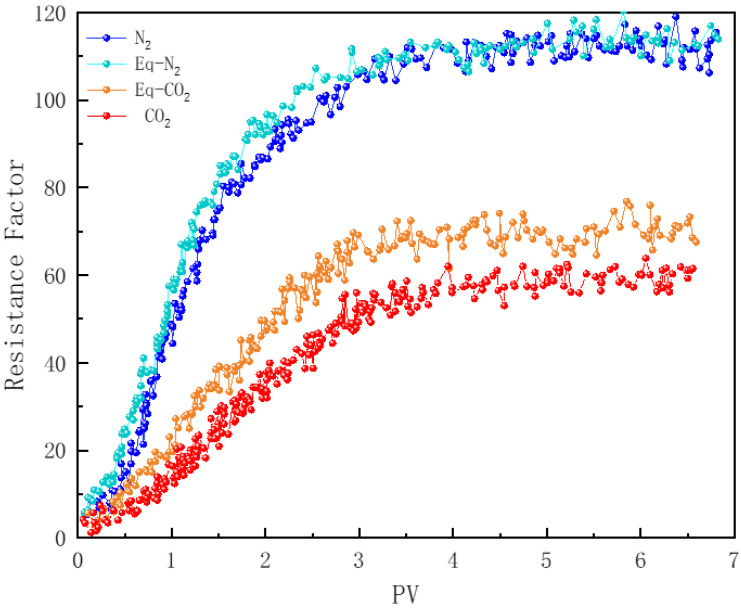
Comparing equilibrated and non-equilibrated PEF.

The mass transfer between gas and water can significantly impact foam stability. N_2_ has a relatively low solubility in water, whereas CO_2_’s solubility is much higher [78]. Although it has been demonstrated that the dissolution and diffusion of CO_2_ in the reservoir lead to a reduction in CO_2_ foam plugging strength, there is limited research on this phenomenon [26]. Core samples numbered 2 and 4 were each injected with foam twice. Initially, foam generated from a non-equilibrated foaming solution with gas was injected into the core. After flushing with formation water, foam generated from a foaming solution equilibrated at the test temperature with gas was reinjected. Figure 16 shows the resistance factors of foam flow within the cores. For N_2_, the effect of gas dissolution on foam strength is negligible, whereas the dissolution of CO_2_ significantly reduces the foam’s plugging effectiveness. Under the experimental conditions, the dissolution of CO_2_ resulted in a reduction in the resistance factor by approximately 20%.

## 4. Conclusions

Here are the conclusions from the study:

(1) Increasing the concentration of CO_2_ in the gas phase significantly enhances the foaming ability of the CHSB surfactant, while increasing the proportion of N_2_ improves the stability of the foam. This phenomenon is mainly attributed to the lower interfacial tension between CO_2_ and water. The stability of the foam is influenced by the diffusion rate of gas through the liquid film and the liquid drainage rate from the film.
(2) In a CO_2_-dominant environment, the stability of the foam can be significantly enhanced by appropriately adjusting the gas composition, adding polymers to reinforce the foam, lowering the application temperature of the foam system, and increasing the gas pressure.
(3) The behavior of foam in porous media differs from that of bulk foam. This difference is primarily due to the significant impact of liquid drainage on the stability of bulk foam, while, in porous media, the coalescence of the foam caused by gas diffusion is more pronounced.
(4) The CHSB foam reinforced with Z364 polymer can effectively control fluid mobility in porous media at temperatures as high as 200 °C. Reducing the CO_2_ content in the gas phase can further enhance the blocking performance of the foam in porous media.

## Figures and Tables

**Table 1:** Core experiment parameters.

No.	Permeability/mD	Porosity/%	Temperature/°C	Back Pressure /MPa	Solution	Gas
1	519	27.67	100	6	PEF	100% N_2_
2	528.7	30.14	150	6	PEF	100% N_2_
3	496.8	29.88	150	6	PEF	50% N_2+_ 50% CO_2_
4	510.1	27.85	150	6	PEF	100% CO_2_
5	506.8	30.91	150	6	0.4% CHSB	100% N_2_
6	524.1	29.96	200	6	PEF	100% N_2_
7	535.3	30.52	150	3	PEF	100% N_2_
8	493.7	27.36	150	15	PEF	100% N_2_

## Data Availability

Data are contained within the article.
